# Mandible segmentation from CT data for virtual surgical planning using an augmented two-stepped convolutional neural network

**DOI:** 10.1007/s11548-022-02830-w

**Published:** 2023-01-13

**Authors:** Tobias Pankert, Hyun Lee, Florian Peters, Frank Hölzle, Ali Modabber, Stefan Raith

**Affiliations:** Department of Oral and Maxillofacial Surgery, RWTH Aachen University Hospital, Aachen, Germany

**Keywords:** Automated surgical planning, 3D-Unet, Medical image segmentation, Mandible segmentation, Anatomical curvature, CT segmentation, Artifact-free segmentation, Data augmentation

## Abstract

**Purpose:**

For computer-aided planning of facial bony surgery, the creation of high-resolution 3D-models of the bones by segmenting volume imaging data is a labor-intensive step, especially as metal dental inlays or implants cause severe artifacts that reduce the quality of the computer-tomographic imaging data. This study provides a method to segment accurate, artifact-free 3D surface models of mandibles from CT data using convolutional neural networks.

**Methods:**

The presented approach cascades two independently trained 3D-U-Nets to perform accurate segmentations of the mandible bone from full resolution CT images. The networks are trained in different settings using three different loss functions and a data augmentation pipeline. Training and evaluation datasets consist of manually segmented CT images from 307 dentate and edentulous individuals, partly with heavy imaging artifacts. The accuracy of the models is measured using overlap-based, surface-based and anatomical-curvature-based metrics.

**Results:**

Our approach produces high-resolution segmentations of the mandibles, coping with severe imaging artifacts in the CT imaging data. The use of the two-stepped approach yields highly significant improvements to the prediction accuracies. The best models achieve a Dice coefficient of 94.824% and an average surface distance of 0.31 mm on our test dataset.

**Conclusion:**

The use of two cascaded U-Net allows high-resolution predictions for small regions of interest in the imaging data. The proposed method is fast and allows a user-independent image segmentation, producing objective and repeatable results that can be used in automated surgical planning procedures.

## Introduction

Patient-specific computer planning has become an indispensable tool for complex surgeries in the cranio-maxillofacial domain such as mandibular reconstructions [1, 2]. Large parts of these planning procedures can be automated to standardize the treatment and to overcome operator dependence in the planning stage [3–5]. However, the upstream process of capturing the correct geometrical surfaces of the relevant anatomical compartments from medical imaging data has only been scarcely investigated, even though it is paramount for the accuracy and the success of any subsequent virtual surgical planning.

Computed tomography (CT) scans of the head are often strongly affected by imaging artifacts caused by metal inlays or implants. Manually removing these artifacts is a time-consuming and labor-intensive process, amplifying the need for automated segmentations.

In many clinical domains, medical image segmentation has been disrupted by recent approaches using different techniques, synoptically referred to as artificial intelligence. The U-Net architecture [6] based on a fully convolutional network for semantic segmentation [7] has shown to outperform competing algorithms to segment various body parts including lung, liver, bone and pathological region such tumor [8–10]. The segmentation of the mandibular bone was the topic of recent publications and segmentation challenges [11, 12]. The approaches in these works are promising and show partly convincing results.

However, none of the approaches have yet focused on the assessment of the segmentations with the aim of usage in a clinical process chain of surgical planning for facial reconstruction. For the use of segmented data of the mandible in virtual surgical planning, specialized criteria need to be considered, as not all anatomical structures of the mandibular bone are equally important in the scope of reconstructive surgery. Due to capacity restrictions of the computing devices, processing full resolution, three-dimensional CT scans by Convolutional Neural Networks (CNNs) is not feasible.

Thus, we aimed to develop a fully automated pipeline to perform accurate segmentations of the mandible from CT imaging, that can be used for surgical planning of mandibular reconstructions while retaining a feasible computational effort to allow use in clinical routine. The models are trained using a large real-world dataset of mandibles, originating from the context of surgical planning. In this pipeline, we use data augmentation, and we evaluate three different loss function, i.e., the Dice loss, Tversky loss [13] and Focal Tversky loss [14]. For the evaluation of the accuracy, we introduce the caudolateral curve distance, an observer-independent, anatomically inspired metric that respects bony curvatures [4] and thus relates the segmentation’s accuracy better to clinical requirements as compared to established generic metrics such as Dice coefficient or 3D surface deviations with the aim of producing data for subsequent surgical planning.

## Material and methods

**Fig. 1:**
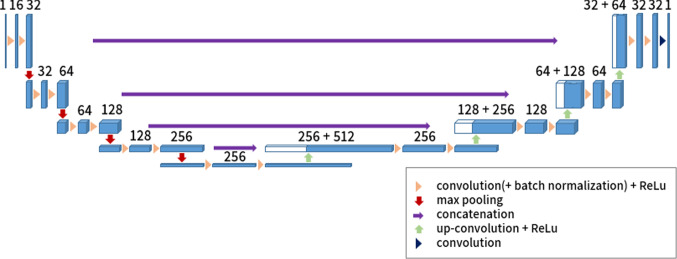
The 3D-U-Net architecture used in this study. All convolutions have kernel sizes of 3 × 3 × 3 while all max-pooling and up-convolution operations have kernel sizes of 2 × 2 × 2

For the semantic segmentation of volumetric imaging data, we use a 3D-U-Net architecture based on [15] and as depicted in Fig. 1.

### Two-step approach

**Fig. 2:**
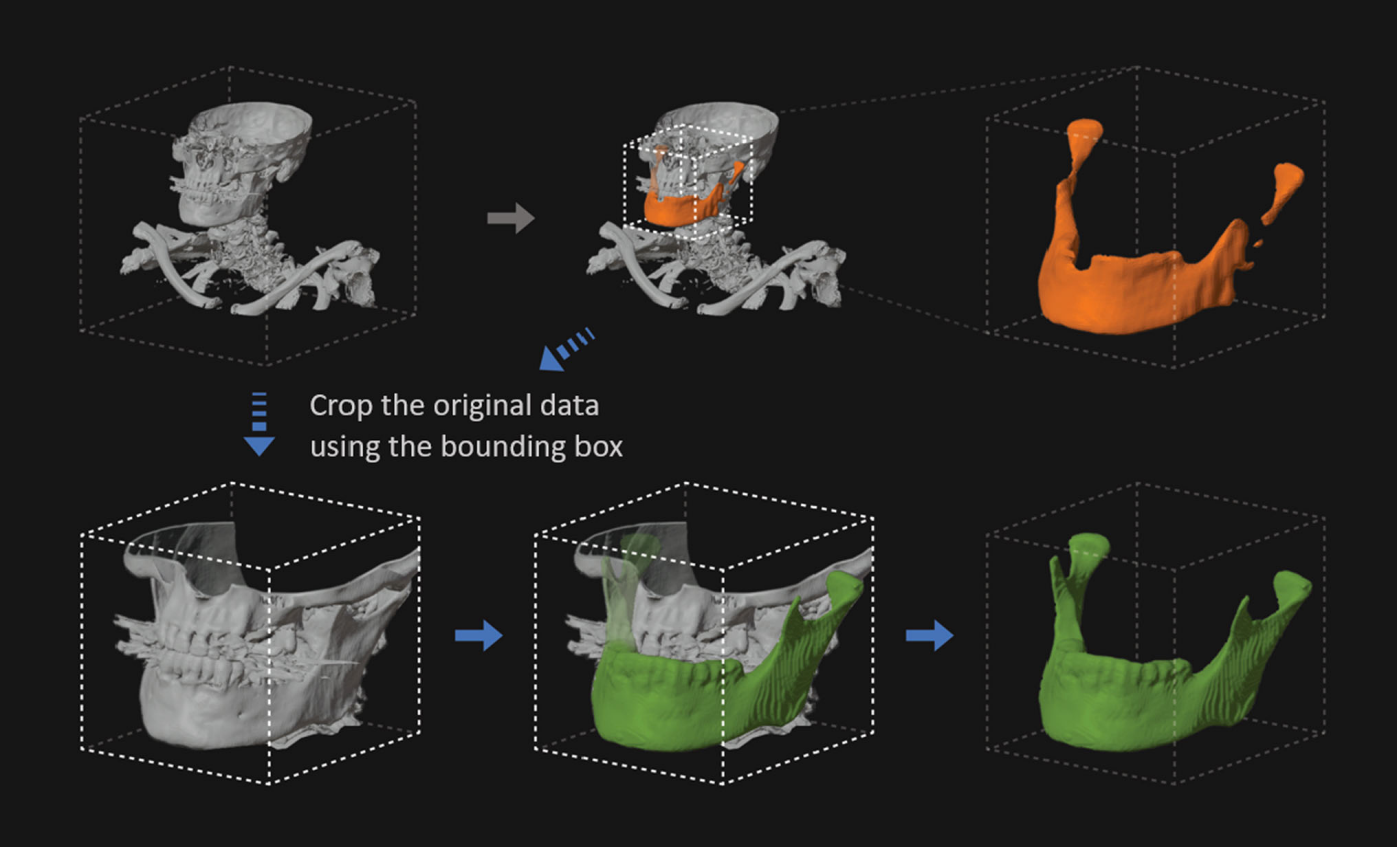
Suggested two-step approach of this study consisting of a first step for localization of the region of interest using a low-resolution prediction (orange) and a second step for a refined prediction (green) (color figure online)

The input sizes of three-dimensional CNNs are limited by capacity restrictions of the computing devices. To overcome this limitation, we propose a two-step approach that uses the result of a first step, low-resolution segmentation to define a bounding box for a second segmentation step that takes into account only the actual area of interest as detected by the first run [16] as depicted in Fig. 2. Thus, a first U-Net was trained on the whole input image data down-sampled to the common input size of 144 × 144 × 144 voxels. For the training of a second U-Net, the full resolution input images were cropped to the bounding boxes of the ground truth segmentations and also sampled to the resolution of 144 × 144 × 144 voxels. Apart from the different training data, both networks were trained with identical parameters.

### Data preprocessing and augmentation

The input CT images are stored with voxel values representing the local radio-density expressed in Hounsfield units (HU). While the representable range depends on the specific implementation of the CT manufacturer, it usually covers at least values from  − 1024 HU to 3071 HU.

For this study, the Hounsfield units were clipped to the range of  − 1024–3071 and mapped linearly to floating point numbers ranging between 0 and 1. Since values above 2000 are nearly exclusively reserved to artificial materials like metals, the remaining range covers all relevant information regarding bones and tissue in the input images while still being able to distinguish them from foreign bodies like implants, inlays, or osteosynthesis materials [17]. For the training of the first step model, the three-dimensional images were then down-sampled to a size of 144 × 144 × x144 voxels using a third-degree spline interpolation.

For the second step model, the images were cropped to the padded bounding boxes of the ground truths, before being resampled again to a size of 144 × 144 × 144 voxels.

To reduce overfitting of the model, we applied several methods of data augmentation during the training pipeline [18]. Mirroring, spatial translations, blurring, additive Gaussian noise, down-sampling, scaling, rotations and elastic deformations were used as provided by the *batchgenerators* library [19].

### Loss functions

Since the field of view of the underlying CT scans usually contains the entire head and neck region, the mandible only occupies a relatively small part of the imaging data. Thus, three loss functions that are beneficial in dealing with this imbalance issue were chosen for training of the networks.

The Dice loss (DL) function is defined as (1–Dice Coefficient (DC)) and measures the overlap of the ground truth and the prediction in relation to the sum of both volumes. Since the background is not considered, weighting factors are not necessary to establish the right balance between foreground and background voxels [20].

The dice coefficient is defined as.

$$ {\text{DC}} = \frac{{2{\text{TP}}}}{{2{\text{TP}} + {\text{FN}} + {\text{FP}}}}; $$

where TP (true positives) is the number of overlapping voxels from the ground truth and the prediction and FN (false negatives) and FP (false positives) are the numbers of voxels present either only in the ground truth (FN) or prediction (FP), respectively.

The Tversky loss (TL) function, derived from the Tversky Index (TI) [21] is extending the Dice loss with the weighting factors *α* and *β* to control the magnitude of penalties for false positives and false negatives, respectively:

$$ {\text{TL}} = 1 - {\text{TI}} = 1 - \left( {\frac{{{\text{TP}}}}{{{\text{TP}} + \alpha {\text{FN}} + \beta {\text{FP}}}}} \right). $$

For *α* + *β* = 1 and *β* > *α*, it weights sensitivity higher than precision by emphasizing false negatives more.

The Focal Tversky Loss (FTL) uses the parameter *γ* to add nonlinearity to the Tversky loss. This nonlinearity allows for controlling how the loss behaves at different Tversky Indices.

$$ {\text{FTL}} = \left( {1 - TI} \right)^{\gamma } $$

By setting *γ* > 1, a higher loss gradient is achieved for harder examples of TI < 0.5. This enables the model to focus on learning the harder examples such as highly imbalanced data which usually get smaller TI [14]. For this study, we selected *α* = 0.3, *β* = 0.7 and *γ* = 4/3 based on the values proposed by [14].

### Post-processing

Before transforming the voxel-based network prediction to three-dimensional surfaces for subsequent evaluation, a 3D binary erosion and a Gaussian smoothing were applied to the network output to remove small outliers and smooth the surfaces. The erosion was required since the conversion from surface data to voxel-based training data produces slightly dilated structures. Finally, the 3D voxel-based data were converted to surface models by using the marching cubes algorithm [22].

### Evaluation metrics

For quantification of accordance between automated segmentations and ground truths, five different metrics were used that are described in the following.

The Dice similarity coefficient (DSC), as defined in Sect. "Loss functions," is a widely used generic metric for accordance between three-dimensional closed objects, often used to measure the performance of computerized segmentation in medical imaging [23].

The average surface distance (ASD) describes a projection of the test surface to a reference surface. Advantageous in this metric is that it is respecting outliers in a meaningful way, as the distance of the potential outliers from the reference surface is considered. Since we used a one-directional surface distance, the computed distances would differ for switched tests and reference objects.

The Hausdorff distance is a metric defined as the maximum of the 3D surface distance and the derived 95%-Hausdorff distance (95HD) is defined as the 95th percentile of all surface deviations, thus being more robust against outliers [24].

In previous works, a caudolateral curve was introduced that describes the caudolateral demarcation of the mandible and may be used for the automated planning of facial reconstructions with bone transplants [4]. In the present work, this curve was automatically generated by projecting a curve that is individualized by automatically detected landmark points on the mandible [25]. For the caudolateral curve distance (CCD), the 3D surface distance is calculated between the anatomical curves of the ground truth of the ground truths and the predictions. This metric only considers connected parts of the mesh and is thus very vulnerable to disconnected predictions.

Lastly, since we use the prediction from the first step model to compute a bounding box of the mandible to crop the input image to, the intersection-over-union of the bounding boxes (BB IoU) of the ground truths and predictions is used to assess the quality of the predictions regarding its usefulness for the cropping step.

### Patient data

The data used in this study comprised a total of 307 subjects from our institution who had undergone CT scans of the head and neck region for clinical indications in the time between 2007 and 2015. Criteria for exclusion in primary data collection were bone fracture and history of facial trauma, bone transplant or augmentation, and bony malformation due to syndromes or other congenital craniofacial anomalies or mandibular tumors. Dental status was not chosen as a selection criterion, thus the data comprises fully and partial dentate, as well as edentulous mandibles. The pixel spacing varied from 0.2 to 0.9 mm in both lateral axes. In the vertical axis, the variation ranged from 0.1 to 4 mm but most of the data had a slice distance of 0.7 mm. Patients with any evidence of skeletal mandibular disease and malformations were excluded based on judgments by an experienced cranio-maxillofacial surgeon. For conformity with legislation on data protection, all subjects' data were pseudonymized. No consecutive scans of the same patients were included in the dataset to avoid data redundancy. 63.5% (195) of the patients were male and 36.5% (112) female, the mean age was 63 years. No data on ethnic origin of the subjects were available.

**Fig. 3:**
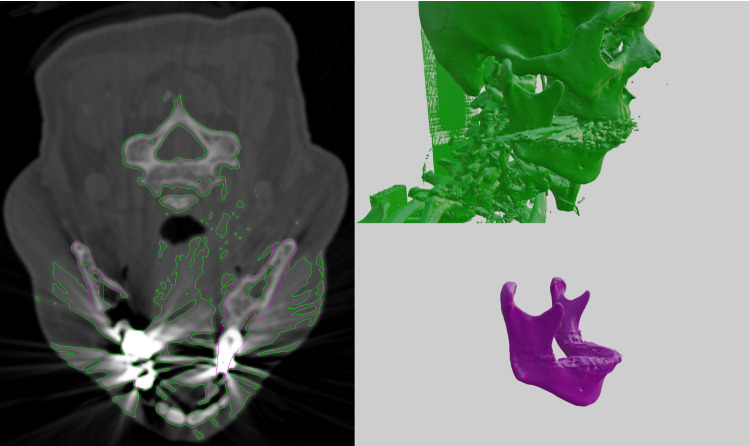
Imaging artifacts in a CT acquisition. On the left: Projection of a threshold-based segmentation with a threshold of 250 HU (green) and of the ground truth segmentation (magenta) on a CT slice. On the right: 3D-renders of the same segmentations. The ray-shaped artifacts are clearly visible (color figure online)

The ground truths were prepared by cranio-maxillofacial surgeons using a semi-automated process in the software Mimics 14 (Materialise Inc., Leuven, Belgium). Based on threshold-based pre-segmentations, the surgeons labeled the CT images in three axes in space to triangulate 3D geometric surface information of each mandible in STL format using a marching cubes algorithm. The segmentations included the mandible bones as well as the mandibular teeth, imaging artifacts were manually removed at the discretion of the operator [Fig. 3], as previously described [26]. For the training of the network, the 3D surfaces were converted back to binary volumetric data.

### Study design

The data were divided into a training set of 248 samples, a validation set of 30 samples and a test set of 29 samples. We used a fully random selection to divide our set of data into these three groups. For all models used for the generation of the results below, the same distributions were used. The models were trained on the training and validation sets for the first and second step independently using each in combination with either the Dice loss, Tversky loss, or Focal Tversky loss functions, respectively, while the loss functions were the same for both corresponding steps.

All predictions generated with the different models were evaluated with the metrics described in Sect. "Evaluation metrics."

### Statistical evaluation

To evaluate the statistical significance of the results, multiple dependent t-tests for paired samples were performed. For each of the five evaluated metrics, tests were performed to determine the significance for the use of the two-step approach as well as pair-wise comparisons of the three loss functions for both the first and the second step. The resulting p-values from the comparisons between the loss functions were corrected, independently for each evaluated metric, using the Holm-Šídák method [27]. Depending on the resulting p-values, results were classified as “not significant” (*p* ≥ 0.05), “significant” (0.01 ≤ *p* < 0.05), “very significant” (0.001 ≤ *p* < 0.01) or “highly significant” (*p* < 0.001).

## Results

### Qualitative results

**Fig. 4:**
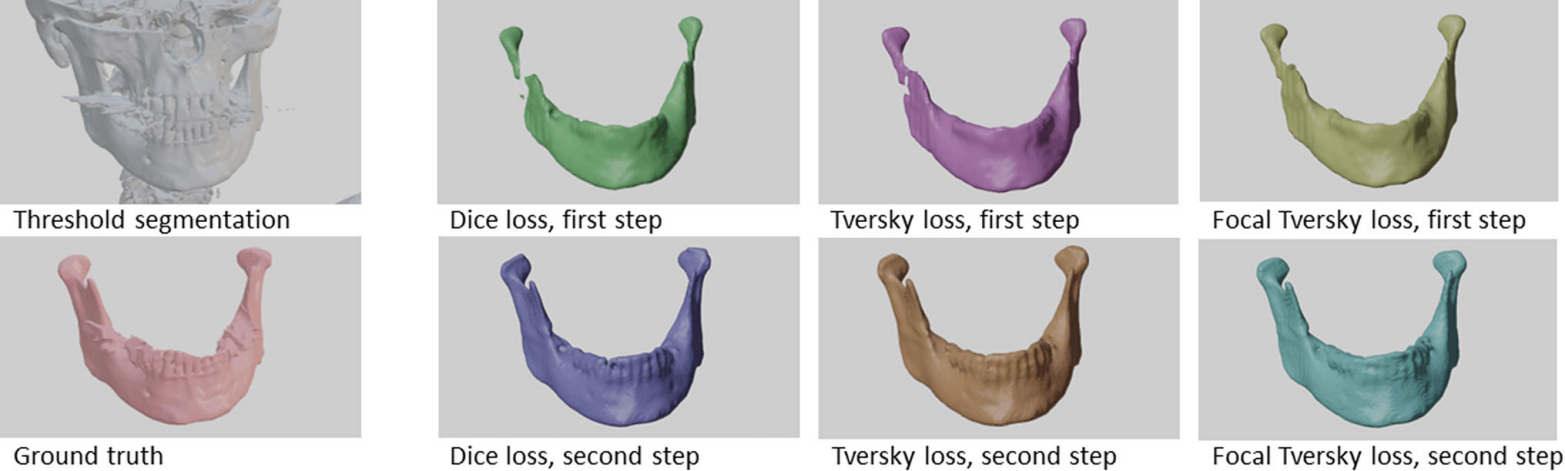
Threshold segmentation, ground truth and predictions from all si x evaluated models for an individual from the test dataset

Predictions from all different models for one exemplary patient from the test dataset are seen in Fig. 4. All models are able to remove the imaging artifacts present in the imaging data.

**Fig. 5:**
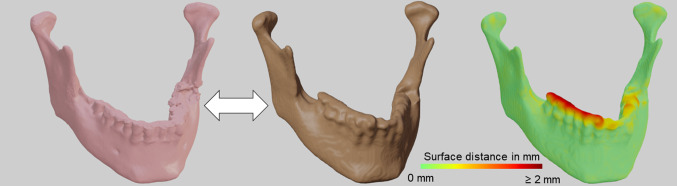
Projection of the surface distance (right) between a prediction from a two-step Tversky loss model (middle) and the corresponding ground truth (left)

**Fig. 6:**
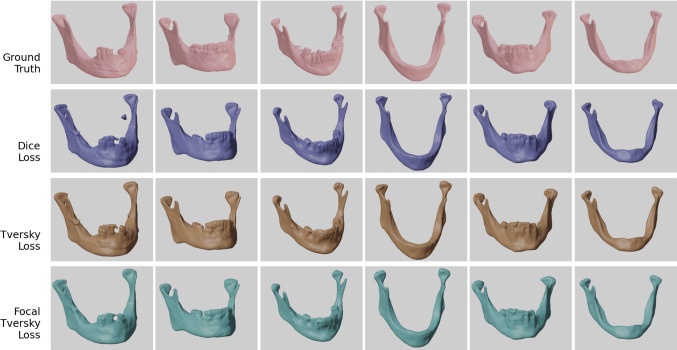
Predictions from all two-step models and the corresponding ground truths for six patients from the test dataset

While the single step predictions show to have problems identifying narrow parts of the bone, especially the condylar and the coronoid processes, overall good predictions with highly accurate segmentations for most parts of the bone are achieved by all two-step models. However, problematic regions for segmentation were found at the teeth and condyles [Fig. 5]. For the single step predictions, the TL and FTL models provide visibly better results than the DL model [Fig. 4], while for the two-step setup, a visual distinction between the shapes predicted from the different model is the choice of loss functions has less influence on the shapes [Fig. 6].

In general, toothless mandibles are segmented very accurately overall, while dentate mandibles showed imprecisions, especially at the tooth cusps [Fig. 6].

### Quantitative results

The quantitative evaluation of the trained models on the test dataset is seen in Table 1, the statistical evaluation in Table 2.

**Table 1:** Evaluation of all models based on average surface distance (ASD), 95% Hausdorff distance (95HD), Caudolateral curve distance (CCD), Dice score (DSC) and bounding box overlap (BB IoU)

Model	ASD (mm)	95HD (mm)	CCD (mm)	DSC (%)	BB IoU (%)
Single step + dice loss	1.34 ± 0.27	2.58 ± 0.45	9.44 ± 4.95	92.95 ± 1.89	81.89 ± 5.81
Single step + Tversky loss	1.09 ± 0.23	2.28 ± 0.42	6.94 ± 4.93	91.75 ± 1.58	85.63 ± 4.63
Single step + focal Tversky loss	1.03 ± 0.20	2.19 ± 0.38	5.94 ± 4.13	91.54 ± 1.64	85.76 ± 4.46
Two-step + dice loss	0.36 ± 0.11	0.90 ± 0.31	1.24 ± 0.77	94.82 ± 1.91	93.38 ± 2.79
Two-step + Tversky loss	0.31 ± 0.09	0.96 ± 0.44	1.30 ± 1.02	93.87 ± 1.71	94.74 ± 2.12
Two-step + focal Tversky loss	0.35 ± 0.08	0.97 ± 0.31	1.71 ± 1.39	93.94 ± 1.79	94.85 ± 3.02

**Table 2:** Statistical evaluation using related *t*-tests and Holm-Šídák correction

Comparison	ASD	95HD	CCD	DSC	BB IoU
Single step $\leftrightarrow$ two-step	< < <	< < <	< < <	< < <	< < <
DL$\leftrightarrow$ TL (single step)	< < <	< < <	< < <	> > >	< < <
DL $\leftrightarrow$ TL (two-step)	< < <			> > >	< < <
DL $\leftrightarrow$ FTL (single step)	< < <	< < <	< < <	> > >	< < <
DL $\leftrightarrow$ FTL (two-step)		>		> > >	< < <
TL $\leftrightarrow$ FTL (single step)	< < <	< <		> >	
TL $\leftrightarrow$ FTL (two-step)	> > >				

“ > ” indicates the left side of the comparison performed better and “ < ” that the right side performed better

(< / >) indicate “significant” differences, (< < / > >) “very significant” differences and (< <  < / >  > >) “highly significant” differences

#### Single step

For the single step setup, the use of asymmetric loss functions (Tversky loss and Focal Tversky loss) provides highly significantly better results than the use of the Dice loss for all regarded metrics except the Dice score itself, indicating an advantage of these losses for imbalanced sizes of labeled and unlabeled regions. The ASD and 95%HD are slightly better for the first step FTL model than for the TL, while it’s the opposite for the Dice score. All single step models experience high errors for the caudolateral curve distance in combination with high standard deviations. Since the models failed to predict a connected mandible for many subjects from the test dataset, the correct calculation of the caudolateral curve for these examples fails. In comparison with this metric, the average surface distance and the 95% Hausdorff distance are usually much lower, with an average surface distance of below 1.4 mm and a 95% Hausdorff distance of below 2.6 mm for all regarded models. The intersection-over-union of the bounding boxes of the prediction and the ground truth are all within the range of 81–86%, indicating a good basis for a cropping step.

#### Two-step approach

For all metrics except the Dice score, a combination of the second step model with a first step model using the same training configuration was used, the Dice score was computed based on the cropping from the ground truth data.

In contrast to the single step approach, the differences between the different loss functions are much less pronounced for the two-step approach. The Tversky loss is highly significantly better than the Focal Tversky loss for the average surface distance, while for the other metrics all differences are insignificant. The Dice loss performs highly significantly better than both other loss functions for the Dice coefficient but is worse than them for the surface distance. For the caudolateral curve distance, the differences between the loss functions are insignificant.

All models yield an average surface distance below 0.4 mm, a 95% Hausdorff distance below 1.0 mm and a caudolateral curve distance below 1.8 mm. The caudolateral curve distance is not only much smaller than for the single step results, but also has a much lower standard deviation.

Overall, the two-step approach vastly and highly significantly outperforms the single step approach in all configurations. While the dice scores indicate relatively good results for the single step segmentations, the surface-based metrics all show how the second step significantly increases the accuracy of the model.

The models reach accuracies in surface distances in the orders of magnitude of the underlying CT voxel spacing.

## Discussion

The approach described in this paper could demonstrate its applicability for the given task of mandible segmentation out of CT data. The accuracy showed to be sufficient for potential subsequent use in surgical planning, e.g., for facial reconstructions.

The first step models benefit highly from the use of the Tversky or Focal Tversky loss functions in comparison with the Dice loss function, this effect showed to be negligible for the full two-step setup. The finding of superior performance of non-symmetric loss functions in the first step and the diminishing of this effect in the second step may be attributed to the fact that labeled and unlabeled regions are more balanced in the latter step.

The two-step approach provided significantly better results than a single step method with the same resolution. The detection of the region of interest in the first step showed to be robust with no false detections in the investigated test dataset.

One of the main benefits of the presented approach is that it enables an efficient and objective way to get accurate segmentations of the mandible for subsequent surgical planning. This is a crucial step in computer-aided operation planning. The fact that our set of training data was derived from a previous study focusing on data acquisition for reconstructive surgical planning, makes the data suitable for a potential application in this specific takt. Providing a fully automated segmentation model requires considerable work, considering the time and effort invested in preparing the training datasets. However, once the trained models are provided, the segmented mandible can be accessed without the tedious and time-consuming processes in manual or semi-automatic segmentation methods [28–30]. On an *NVIDIA Geforce RTX 2080 Ti* running the two-step prediction pipeline on a DICOM dataset takes approximately 31 s.

Since our model operates on high-resolution datasets, it allows segmentation of the mandible directly from the input CT imaging, without any manual interaction. For this study, the input for the second step models was resampled to a uniform size of 144 × 144 × 144 voxels. However, once trained, our model can operate at larger or smaller resolutions as well, allowing to skip the resampling step to obtain predictions in the original resolution of the CT images.

Our proposed pipeline uses the ground truth-based cropping only for the training pipeline. For the evaluation as well as for a potential application in clinical practice the second step would crop the field of view based on the result from the first step. The real-world application will thus not rely on the presence of a ground truth segmentation.

In the recent review paper on automatic segmentation of mandible by Qui et al., eleven studies of deep learning-based models with a two-step approach were compared [12]. [31] achieved a DSC of 93.12% and a 95HD of 2.48 mm on their In-house dataset as well as a DSC of 95.00% on the publicly available PDDCA dataset while Dijk et al. achieved a DSC of 94.00% and a 95HD of 1.3 mm on their In-House dataset from 693 patients. Our two-step model with Dice loss achieves comparable performances (DSC of 94.82%, 95HD of 0.9 mm).

In [32], the authors propose an automated segmentation based on a prior shape model to segment CBCT scans affected by metal artifacts. On their in-house dataset from 59 patients, they achieve a DSC of 95.35%, an ASD of 0.99 mm and a 95HD of 2.57 mm. 3D network strategies or attention strategies are promising strategies that have been tried in deep learning-based mandible segmentation. [33] used 3D network strategies to achieve a DSC of 95.70% on the PDDCA dataset. An automated model with attention strategies developed by Gou et al. also used the PDDCA dataset [34]. It showed a DSC of 94.00%, an ASD of 0.47 (± 0.11) mm and 1.40 (± 0.02) mm of 95HD.

Our approach is less prone to outliers, in contrast to other approaches, e.g., the work of Qiu et al. [35]. However, there are inaccuracies in the region of dental occlusion already present in the available ground truth data, in which the separation is often not perfect either. As the present study is focused on the segmentation of mandible for the planning of bony reconstructions, the region of the teeth is less important. Additionally, our method can accurately segment mandibles affected by heavy imaging artifacts.

However, all these comparisons bear the limitation, that none of the referenced studies used our exact set of training data. Thus, to allow a more direct comparison with other studies, it is planned to apply the proposed method to a public domain dataset. Another envisioned approach to enable a fair comparison to other publications will be to use the implementations of other researchers and evaluate those with the dataset presented in this study. This approach would have the advantage to have the specific close connection of our dataset to the application in surgical planning but is restricted by the open availability of the competing implementations.

In conclusion, we provided a segmentation pipeline suitable for the clinical application in the scope of facial reconstructions surgery planning, as indicated by evaluation of the accuracy with various metrics, including the caudolateral curves that are specifically developed for surgical planning in reconstructive surgery of the mandible. The pipeline reaches very high accuracies in relation to manually segmented ground truth data on our real-world dataset that is for most metrics superior to the results achieved with previously published approaches, based on roughly comparable data. We were to the best of our knowledge the first to demonstrate the positive influence of the Tversky and Focal Tversky loss functions for segmentation of facial bones in the presence of imaging artifacts, especially on the first part of our two-step approach.

In future work, the method will be extended to the maxilla and midface region with the aim of providing an automated pipeline for the reconstruction of the bony midface. The usage of the presented approach in an automated pipeline for surgical planning of mandibular reconstructions may be envisioned, but regulatory constraints do apply as software that is used in the process chain of surgical planning is considered as a medical product.
